# Effects of underweight and overweight on mortality in patients with pulmonary tuberculosis

**DOI:** 10.3389/fpubh.2023.1236099

**Published:** 2023-09-19

**Authors:** Jinsoo Min, Ju Sang Kim, Hyung Woo Kim, Yousang Ko, Jee Youn Oh, Yun-Jeong Jeong, Eun Hye Lee, Bumhee Yang, Ki Man Lee, Joong Hyun Ahn, Jin Woo Kim, Yong Il Hwang, Sung Soon Lee, Jae Seuk Park, Hyeon-Kyoung Koo

**Affiliations:** ^1^Division of Pulmonary and Critical Care Medicine, Department of Internal Medicine, Seoul St. Mary's Hospital, College of Medicine, The Catholic University of Korea, Seoul, Republic of Korea; ^2^Division of Pulmonary and Critical Care Medicine, Department of Internal Medicine, Incheon St. Mary's Hospital, College of Medicine, The Catholic University of Korea, Seoul, Republic of Korea; ^3^Division of Pulmonary, Allergy and Critical Care Medicine, Department of Internal Medicine, Kangdong Sacred Heart Hospital, Hallym University College of Medicine, Seoul, Republic of Korea; ^4^Division of Pulmonary, Allergy, and Critical Care Medicine, Department of Internal Medicine, Korea University Guro Hospital, Korea University College of Medicine, Seoul, Republic of Korea; ^5^Division of Pulmonary and Critical Care Medicine, Department of Internal Medicine, Dongguk University Ilsan Hospital, Goyang, Republic of Korea; ^6^Division of Pulmonology, Allergy and Critical Care Medicine, Department of Internal Medicine, Yongin Severance Hospital, Yonsei University College of Medicine, Seoul, Republic of Korea; ^7^Division of Pulmonary and Critical Care Medicine, Department of Internal Medicine, Chungbuk National University Hospital, Chungbuk National University College of Medicine, Cheongju, Republic of Korea; ^8^Division of Pulmonary and Critical Care Medicine, Department of Internal Medicine, Uijeongbu St. Mary's Hospital, College of Medicine, The Catholic University of Korea, Uijeongbu, Republic of Korea; ^9^Department of Pulmonary, Allergy and Critical Care Medicine, Hallym University Sacred Heart Hospital, Anyang, Republic of Korea; ^10^Division of Pulmonary and Critical Care Medicine, Department of Internal Medicine, Ilsan Paik Hospital, Inje University College of Medicine, Goyang, Republic of Korea; ^11^Division of Pulmonary Medicine, Department of Internal Medicine, Dankook University College of Medicine, Cheonan, Republic of Korea

**Keywords:** tuberculosis, undernutrition, nutrition, death, mortality, body mass index

## Abstract

**Background:**

Poor nutrition increases disease severity and mortality in patients with tuberculosis (TB). There are gaps in our understanding of the effects of being underweight or overweight on TB in relation to sex.

**Methods:**

We generated a nationwide TB registry database and assessed the effects of body mass index (BMI) on mortality in patients with pulmonary TB. The cause of death was further classified as TB-related or non-TB-related deaths. First, logistic regression analysis was performed to assess the association between BMI (a continuous variable) and mortality, and subgroup analyses of the multivariable logistic regression model were performed separately in male and female patients. Second, we categorized BMI into three groups: underweight, normal weight, and overweight, and assessed the impact of being underweight or overweight on mortality with reference to normal weight.

**Results:**

Among 9,721 patients with pulmonary TB, the mean BMI was 21.3 ± 3.4; 1,927 (19.8%) were underweight, and 2,829 (29.1%) were overweight. In multivariable logistic regression analysis, mortality was significantly increased with the decrement of BMI (adjusted odds ratio [aOR] = 0.893, 95% confidence interval [CI] = 0.875–0.911). In subgroup analyses, underweight patients had significantly higher odds of mortality, especially TB-related deaths (aOR = 2.057, 95% CI = 1.546–2.735). The association with mortality and male patients was higher (aOR = 2.078, 95% CI = 1.717–2.514), compared with female patients (aOR = 1.724, 95% CI = 1.332–2.231). Being overweight had a significant protective effect against TB-related death only in females (aOR = 0.500, 95% CI = 0.268–0.934), whereas its effect on non-TB-related death was observed only in males (aOR = 0.739, 95% CI = 0.587–0.930).

**Conclusion:**

Being underweight was linked to high mortality, whereas being overweight had beneficial effects in patients with pulmonary TB.

## Introduction

Tuberculosis (TB) is a public health problem affecting millions of people worldwide (1). Among several modifiable factors associated with TB, undernutrition is a leading risk factor worldwide (2, 3). Nutritional status, such as body mass index (BMI), is one of the key factors that categorizes the phenotypes in patients with TB (4). Prevalence of being underweight was approximately 3-fold higher in patients with TB (5). Poor nutritional status is associated with increased severity and mortality of TB disease (6, 7). According to a large study conducted in India, each unit increase in BMI at baseline is associated with a 22% reduction in mortality (8).

Despite the economic growth over the last century, the world still faces a heavy burden of malnutrition that crosses geographical and generational boundaries. The United Nations estimated that 118 million more people experienced hunger in 2020 than in 2019 (9). Undernutrition, which is a key social determinant of health, requires urgent attention (10). Climate change threatens food production and distribution systems in regions with high TB burden. Undernutrition and food security issues are likely to become more prevalent owing to the COVID-19 pandemic and ongoing conflicts with increased forced migration (11).

Despite the increasing number of studies on the impact of nutrition on TB, several gaps remain to be addressed (2, 12). First, BMI has been extensively used to assess the nutritional status in clinical practice because of its easy accessibility. However, many prior studies have used either BMI as a continuous variable or dichotomized nutritional status based on a single BMI cut-off (typically 18.5 kg/m^2^) (13). Using BMI as a continuous variable implies its linear relationship with the outcome of interest. A binary BMI variable cannot differentiate among different degrees of nutritional status, which may have various impacts on health outcomes (14). In addition, association between being overweight and treatment outcome has not been extensively studied (7, 15). Therefore, we aimed to evaluate the impact of being underweight and overweight on anti-TB treatment outcome. Furthermore, men and women with TB have different clinical presentation and treatment outcomes. Indeed, significant differences were found regarding location of TB in the body and disease severity between sexes, especially during women's reproductive age (16). In addition, sex differences in anti-TB treatment outcomes were observed (17). Women are particularly vulnerable to nutritional deficiencies because of the cyclical iron loss and childbearing. It is known that poor nutrition in early life reduces females' learning potential, worsens reproductive and maternal health risks, and decreases productivity. However, how the effects of nutritional deficit on TB differ according to sex has not yet been thoroughly investigated. We hypothesized that impact of nutritional status at the baseline on anti-TB treatment outcomes would be different between men and women with TB.

To address these research gaps, we assessed data from the validated nationwide TB cohort registry in Korea and evaluated whether the effects of being underweight or overweight on all-cause mortality vary by sex among patients with pulmonary TB.

## Methods

### Study setting and data collection

**Figure 1:**
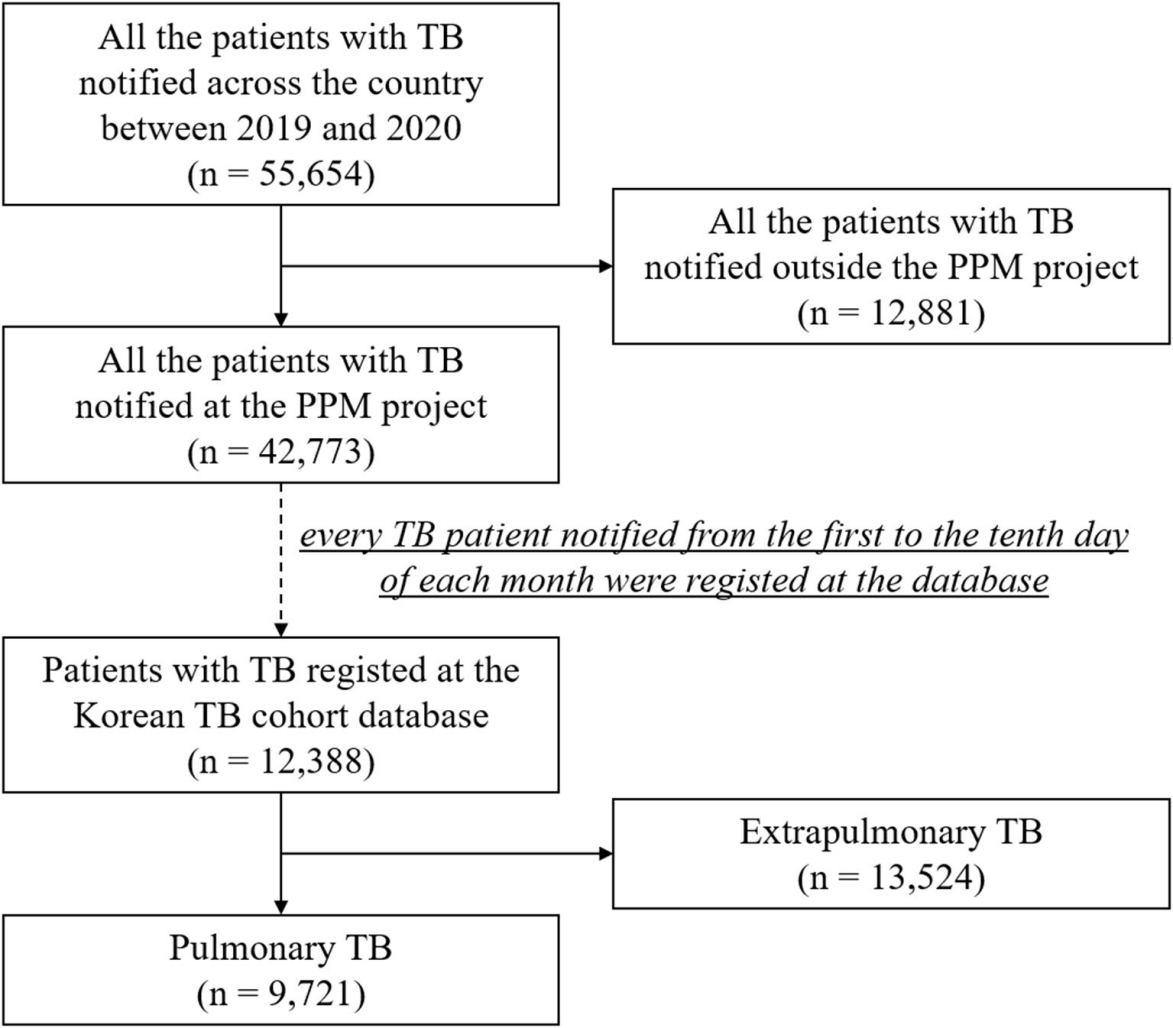
Flow chart of participant enrollment. TB, tuberculosis; PPM, public-private mix.

In the Republic of Korea, a country with an intermediate TB burden (18), physicians are required to report the diagnosis and treatment of TB when it is first diagnosed or suspected. Under the public–private mix (PPM) TB control project (19), all patients are monitored and reported until treatment completion by TB nurse specialists at PPM-participating hospitals. Approximately 77.4% of newly registered TB patients in Korea were treated at PPM hospitals in 2020. Within this project, we constructed *the Korean TB Cohort Database*, a prospective observational registry database, which comprised TB patients notified at PPM-participating hospitals, regardless of age and sex. In this database, every TB patient notified from the 1st to the 10th day of each month was consecutively enrolled across the country. For this study, the only exclusion criterion was extra-pulmonary TB. Finally, the data of patients with pulmonary TB who were notified from January 2019 to December 2020 were retrieved from the national registry database (Figure 1).

Our study was designed as a prospective cohort study, which aimed to assess association between nutritional status and mortality. All the notified TB patients were followed up until the end of anti-TB treatment to confirm their treatment outcomes. The anti-TB treatment outcomes were defined according to the Korean TB guidelines adopted from the World Health Organization. During this period, data of participants were collected by TB specialist nurses using the case report form.

### Variables and outcome definition

Baseline characteristics such as age, sex, BMI, smoking and alcohol history, prior anti-TB treatment history, and coexisting comorbidities were collected as independent variables. We used standard BMI categories for the Asian population: underweight (<18.5 kg/m^2^), normal weight (18.5–22.9 kg/m^2^), and overweight (≥23.0 kg/m^2^). The results of radiographic and microbiological tests, such as sputum acid-fast bacilli (AFB) smear and culture tests, nucleic acid amplification test (NAAT), and drug susceptibility test, were also collected. We collected data on TB-related symptoms using a predefined checklist that included cough, sputum production, dyspnea, chest pain, hemoptysis, fever, general weakness, and body weight loss. If patients denied all the listed symptoms, they were regarded as asymptomatic. The primary outcome of interest was all-cause mortality during anti-TB treatment. The cause of death was further classified as TB-related or non-TB-related based on the medical death certificate.

### Statistical analysis

The baseline characteristics of all enrolled patients are presented as means and standard deviations for continuous variables and as frequencies and percentages for categorical variables. Continuous variables were compared using the *t*-test, and categorical variables were compared using the chi-square test or Fisher's exact test. To explore the associations between initial clinical presentation (TB-related symptoms, positive results of smear, culture, NAAT, and chest radiographic findings of cavitary and bilateral diseases) and BMI among patients with pulmonary TB, their proportions were plotted against BMI (a continuous variable). The adjusted odds ratios (aORs) for four baseline severity indices (symptom presence, AFB smear positivity, and cavitary and bilateral disease) were calculated using multivariable logistic regression, which was adjusted for age, sex, current smoking status, heavy alcohol intake, and presence of comorbidities.

To explore the impact of BMI on the mortality (all-cause mortality, TB-related death, and non-TB-related death) among patients with pulmonary TB, their proportions were plotted against the BMI (as a continuous variable) and additionally stratified by sex. The aORs for mortality were calculated using logistic regression. For multivariable analysis, age, sex, current smoking status, heavy alcohol intake, and presence of comorbidities were adjusted. For the interaction analysis between BMI and sex, interaction terms with sex and BMI were further added to the previous model, and subgroup analyses of the multivariable logistic regression model were performed separately in male and female patients. For a sensitivity analysis, we excluded patients with resistance to isoniazid or rifampicin and followed the same analytical process to explore the effect of BMI on mortality in patients with isoniazid- and rifampicin-susceptible pulmonary TB.

We conducted extended analyses to assess the impact of BMI categories on mortality among patients with pulmonary TB. First, we categorized patients according to BMI into three groups–underweight, normal weight, and overweight–and set the normal weight group as a reference for logistic regression analysis. Second, we calculated the ORs for the mortality in the underweight group with respect to the normal weight reference group. Univariable and multivariable logistic regression analyses were performed. Interaction and subgroup analyses of the multivariable analyses stratified by sex were then conducted. Third, the ORs for mortality in the overweight group were calculated using the same analytical process. All statistical analyses were performed using R software (version 4.2.1). A *p* < 0.05 was considered statistically significant.

### Ethics statement

This study was conducted in accordance with the principles of the Declaration of Helsinki. The Institutional Review Board of Ilsan Paik Hospital, Inje University approved the study protocol (ISPAIK 2021-08-012). The Korea Disease Control and Prevention Agency (KDCA) has the authority to hold and analyze surveillance data for public health and research purposes. The KDCA approved the data use and provided the data without personal identification information.

## Results

A total of 9, 721 patients with pulmonary TB, with the mean age of 61.8 years, and 3, 544 (36.5%) female patients were included (Table 1). The mean value of BMI was 21.3 ± 3.4; 1, 927 (19.8%) were underweight, and 2, 829 (29.1%) overweight. Among the enrolled patients, 22.1% were current smokers, 8.2% were heavy drinkers, and 61.1% had underlying comorbidities. The proportions of current smokers and heavy drinkers in the underweight group were 23.2 and 10.8%, respectively, which were significantly higher than those in the other groups. The proportions of resistance to isoniazid and rifampicin were 5.4% (522) and 1.5% (151), respectively.

**Table 1:** Baseline characteristics of enrolled patients with pulmonary tuberculosis.

**Variables**	**Total**	**Underweight**	**Normal weight**	**Overweight**	***P*-value**
	**(*****n** =* **9,721)**	**(*****n** =* **1,927)**	**(*****n** =* **4,965)**	**(*****n** =* **2,829)**	
Age, years	61.8 ± 18.7	63.8 ± 20.0^*^	60.7 ± 19.2	62.5 ± 16.9^*^	< 0.001
Female sex	3, 544 (36.5%)	731 (37.9%)	1878 (37.8%)	935 (33.1%)	< 0.001
Body mass index (kg/m^2^)	21.3 ± 3.4	16.7 ± 1.4^*^	20.8 ± 1.2	25.3 ± 2.2^*^	< 0.001
Smoking status					0.019
Never or ex-smoker	7, 571 (77.9)	1480 (76.8%)	3836 (77.3%)	2255 (79.7%)	
Current smoker	2, 150 (22.1)	447 (23.2%)	1129 (22.7%)	574 (20.3%)^*^	
Drinking status					< 0.001
None or social drinker	8, 926 (91.8)	1718 (89.2%)	4542 (91.5%)	2666 (94.2%)	
Heavy drinker	795 (8.2)	209 (10.8%)^*^	423 (8.5%)	163 (5.8%)^*^	
**Comorbidity**					
Presence of any disease	5, 939 (61.1)	1,171 (60.8%)	2,880 (58.0%)	1,888 (66.7%)^*^	< 0.001
Diabetes	2, 117 (21.8)	352 (18.3%)	1,013 (20.4%)	752 (26.6%)^*^	< 0.001
Chronic lung disease	576 (5.9)	120 (6.2%)	271 (5.5%)	185 (6.5%)	0.125
Chronic heart disease	570 (5.9)	92 (4.8%)	257 (5.2%)	221 (7.8%)^*^	< 0.001
Chronic liver disease	256 (2.6)	51 (2.6%)	132 (2.7%)	73 (2.6%)	0.978
Chronic kidney disease	325 (3.3)	64 (3.3%)	157 (3.2%)	104 (3.7%)	0.478
Chronic neurologic disease	963 (9.9)	257 (13.3%)^*^	498 (10.0%)	208 (7.4%)^*^	< 0.001
Malignancy	879 (9.0)	202 (10.5%)^*^	426 (8.6%)	251 (8.9%)	< 0.001
Autoimmune disease	102 (1.0)	23 (1.2%)	50 (1.0%)	29 (1.0%)	0.784
Gastrectomy	98 (1.0)	35 (1.8%)^*^	49 (1.0%)	14 (0.5%)^*^	< 0.001
**Initial presenting symptoms**					
Yes	6, 284 (64.6)	1,434 (74.4%)	3,159 (63.6%)	1,691 (59.8%)^*^	< 0.001
No	3, 434 (35.3)	493 (25.6%)	1,804 (36.3%)	1,137 (40.2%)	
**Sputum microbiological tests**					
AFB smear positivity	2, 548 (26.2%)	695 (36.1%)^*^	1,288 (25.9%)	565 (20.0%)^*^	< 0.001
NAAT positivity	3, 989 (41.0%)	928 (48.2%)^*^	2,036 (41.0%)	1,025 (36.2%)^*^	< 0.001
AFB culture positivity	5, 173 (53.2%)	1,120 (58.1%)^*^	2,639 (53.2%)	1,414 (50.0%)^*^	< 0.001
**Chest radiograph findings**					
Cavitation	1, 523 (15.7%)	427 (22.2%)^*^	738 (14.9%)	358 (12.7%)^*^	< 0.001
Bilateral infiltrations	2, 999 (30.9%)	801 (41.6%)^*^	1,506 (30.3%)	692 (24.5%)^*^	< 0.001
**DST result**					
INH-resistance	522 (5.4%)	113 (5.9%)	260 (5.2%)	149 (5.3%)	0.560
RIF-resistance	151 (1.5%)	35 (1.8%)	84 (1.7%)	32 (1.1%)	0.091
**Mortality**					
All cause death	1, 101 (11.3%)	371 (19.3%)^*^	498 (10.0%)	232 (8.2%)^*^	< 0.001
TB-related death	244 (2.5%)	95 (4.9%)^*^	107 (2.2%)	42 (1.5%)^*^	< 0.001
Non-TB-related death	837 (8.6%)	265 (13.8%)^*^	394 (7.7%)	188 (6.6%)	< 0.001

TB, tuberculosis; AFB, acid-fast bacilli; NAAT, nucleic acid amplification test; DST, drug susceptibility test; INH, isoniazid; RIF, rifampicin.

* Indicates a statistically significant difference. Standard BMI categories for the Asian population is used to define underweight (< 18.5 kg/m^2^), normal weight (18.5–22.9 kg/m^2^), and overweight (≥23.0 kg/m^2^).

At least one TB-related symptom was observed in 64.6% of the patients. The most common symptom was cough with or without sputum (41.3%), followed by dyspnea (16.0%) (Supplementary Table S1). Cough, with or without sputum, had a negative linear association with BMI (Supplementary Figure S1). The positivity rates for the AFB smear, NAAT, and AFB culture were 26.2, 41.0, and 53.2%, respectively. Cavitary and bilateral lesions on chest radiography were observed in 15.7 and 30.9% of patients, respectively. The proportion of positive microbiological test results was negatively correlated with BMI (Supplementary Figure S2). Similar trends were observed for cavitary and bilateral diseases on chest radiographs. In the multivariable logistic regression analysis, we observed a consistent inverse relationship between BMI and all baseline severity indices: symptom presence (aOR = 0.942, 95% confidence interval [CI] = 0.930–0.954), positive AFB smear test results (aOR = 0.918, 95% CI = 0.905–0.931), cavitary disease (aOR = 0.926, 95% CI = 0.910–0.942), and bilateral disease (aOR = 0.919, 95% CI = 0.907–0.932) (Table 2).

**Table 2:** Effect of body mass index on initial clinical presentation among patients with pulmonary tuberculosis.

	**Univariable analysis**	**Multivariable analysis**
	**cOR (95% CI)**	**aOR (95% CI)**
Symptom presence	0.941 (0.929–0.952)	0.942 (0.930–0.954)
AFB smear positivity	0.913 (0.901–0.926)	0.918 (0.905–0.931)
Cavitary disease	0.928 (0.912–0.943)	0.926 (0.910–0.942)
Bilateral disease	0.917 (0.905–0.930)	0.919 (0.907–0.932)

cOR, crude odds ratio; CI, confidence interval; aOR, adjusted odds ratio; Coef, coefficient; AFB, acid-fast bacilli; TB, tuberculosis. The odds ratios for each variable are calculated using logistic regression. Multivariate logistic regression analysis is adjusted for age, sex, current smoking status, heavy alcohol intake, and the presence of underlying comorbidities.

**Figure 2:**
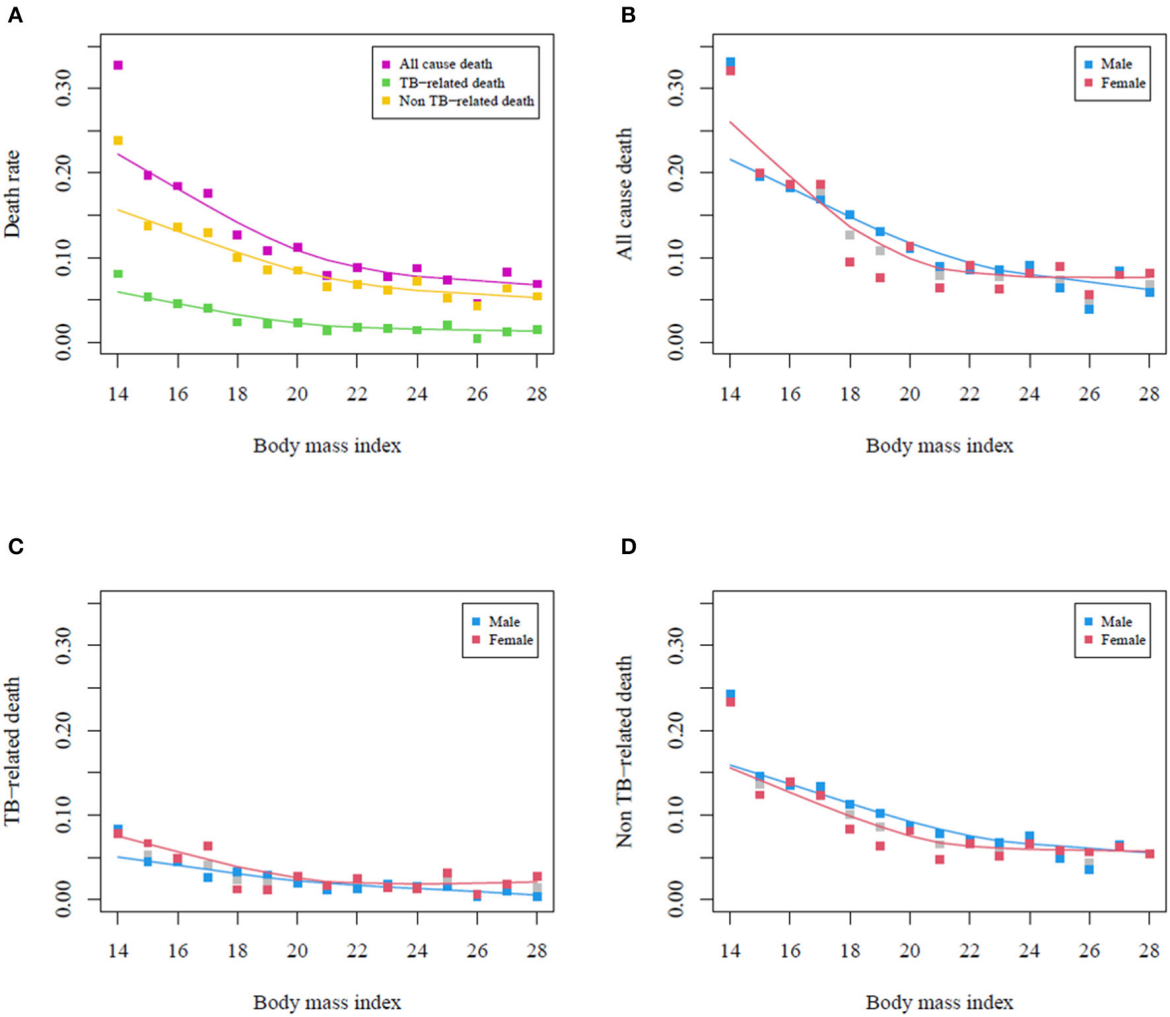
Presence of subtypes of mortality according to body mass index in pulmonary tuberculosis patients. **(A)** All-cause mortality and TB-related and non-TB-related deaths. **(B)** All-cause mortality stratified by sex. **(C)** TB-related death stratified by sex. **(D)** Non-TB-related death stratified by sex. TB, tuberculosis.

During the course of anti-TB treatment, 1, 101 (11.3%) deaths occurred, including 244 (2.5%) TB-related and 837 (8.6%) non-TB-related deaths. The proportion of all-cause mortality was decreased as BMI increased; however, the slopes became less steep after a BMI of 22 kg/m^2^ (Figure 2A), especially among female patients (Figure 2B). Both TB-related and non-TB-related deaths showed similar decreasing trends. At all BMI intervals, female patients had higher rates of TB-related death than male patients did (Figure 2C). In contrast, the non-TB-related death rate was higher in male patients than in female patients (Figure 2D). All-cause mortality was increased with the decrement of BMI (aOR = 0.893, 95% CI = 0.875–0.911) (Table 3). Both TB-related (aOR = 0.878, 95% CI = 0.844–0.914) and non-TB-related (aOR = 0.909, 95% CI = 0.889–0.929) deaths were also increased with the decrement of BMI. We did not identify any interaction between BMI and sex with respect to the mortality rate. After excluding 559 patients with isoniazid- or rifampicin-resistant pulmonary TB, we further conducted a sensitivity analysis to assess association between BMI and mortality among 9, 162 patients with isoniazid- and rifampicin-susceptible pulmonary TB, which revealed the similar findings to those in the original analysis (Table 4).

**Table 3:** Effect of body mass index on mortality among all the enrolled patients with pulmonary tuberculosis.

**Mortality**	**Univariable analysis**	**Multivariable analysis**	**BMI** ^^*^^Sex		**Male**	**Female**
	**cOR (95% CI)**	**aOR (95% CI)**	**Coef**	***P*** **-value**	**aOR (95% CI)**	**aOR (95% CI)**
All cause of death	0.888 (0.871–0.906)	0.893 (0.875–0.911)	0.036	0.087	0.880 (0.858–0.903)	0.916 (0.886–0.946)
TB-related death	0.860 (0.82600.896)	0.878 (0.844–0.914)	0.041	0.314	0.861 (0.817–0.907)	0.905 (0.851–0.962)
Non-TB-related death	0.905 (0.885–0.925)	0.909 (0.889–0.929)	0.034	0.148	0.898 (0.873–0.923)	0.930 (0.8970.965)

BMI, body mass index; cOR, crude odds ratio; CI, confidence interval; aOR, adjusted odds ratio; Coef, coefficient; AFB, acid-fast bacilli; TB, tuberculosis. The odds ratios for each outcome are calculated using logistic regression. Multivariable analysis is adjusted for age, sex, current smoking status, heavy alcohol intake, and presence of underlying comorbidities. BMI is set as a continuous variable.

* Interaction analysis between BMI and sex.

**Table 4:** Sensitivity analysis of the effect of body mass index on mortality among 9,162 patients with isoniazid- and rifampicin-susceptible pulmonary tuberculosis.

**Mortality**	**Univariable analysis**	**Multivariable analysis**	**BMI** ^^*^^Sex		**Male**	**Female**
	**cOR (95% CI)**	**aOR (95% CI)**	**Coef**	***P-*** **value**	**aOR (95% CI)**	**aOR (95% CI)**
All cause of death	0.889 (0.871–0.907)	0.894 (0.875–0.912)	0.038	0.075	0.880 (0.858–0.904)	0.918 (0.887–0.949)
TB-related death	0.864 (0.829–0.899)	0.882 (0.847–0.918)	0.042	0.305	0.864 (0.819–0.911)	0.909 (0.855–0.967)
Non-TB-related death	0.904 (0.884–0.925)	0.908 (0.888–0.929)	0.035	0.141	0.897 (0.871–0.923)	0.930 (0.896–0.966)

BMI, body mass index; cOR, crude odds ratio; CI, confidence interval; aOR, adjusted odds ratio; Coef, coefficient; AFB, acid-fast bacilli; TB, tuberculosis. The odds ratios for each outcome are calculated using logistic regression. Multivariable analysis is adjusted for age, sex, current smoking status, heavy alcohol intake, and presence of underlying comorbidities. BMI is set as a continuous variable.

* Interaction analysis between BMI and sex.

We categorized BMI into three groups and conducted a subgroup analysis to assess the impact of underweight and overweight status on mortality (Table 5). Underweight individuals had significantly higher odds of death, especially TB-related deaths (aOR = 2.057, 95% CI = 1.546–2.735). Its association with male patients was higher (aOR = 2.078, 95% CI = 1.717–2.514), compared with female patients (aOR = 1.724, 95% CI = 1.332–2.231). Being overweight had a negative correlation with both TB-related (aOR = 0.692, 95% CI = 0.452–0.995) and non-TB-related death (aOR = 0.793, 95% CI = 0.659–0.955). However, the negative association between being overweight and TB-related death was statistically significant solely in female patients (aOR = 0.500, 95% CI = 0.268–0.934). In contrast, the protective effects of being overweight on non-TB-related deaths were significant only in male patients (aOR = 0.739, 95% CI = 0.587–0.930).

**Table 5:** Subgroup analysis of the effect of body mass index on mortality among all the enrolled patients with pulmonary tuberculosis.

	**Univariable analysis**	**Multivariable analysis**	**BMI** ^^*^^Sex		**Male**	**Female**
	**cOR (95% CI)**	**aOR (95% CI)**	**Coef**	***P*** **-value**	**aOR (95% CI)**	**aOR (95% CI)**
**(A) Underweight group**						
All cause of death	2.139 (1.848–2.476)	1.951 (1.674–2.274)	−0.171	0.295	2.078 (1.717–2.514)	1.724 (1.332–2.231)
TB-related death	2.354 (1.777–3.120)	2.057 (1.546–2.735)	−0.168	0.567	2.245 (1.543–3.266)	1.765 (1.135–2.746)
Non-TB-related death	1.902 (1.611–2.246)	1.719 (1.446–2.042)	−0.154	0.409	1.812 (1.465–2.241)	1.554 (1.155–2.092)
**(B) Overweight group**						
All cause of death	0.801 (0.681–0.943)	0.755 (0.639–0.893)	0.042	0.813	0.743 (0.604–0.915)	0.781 (0.588–1.037)
TB-related death	0.684 (0.477–0.981)	0.692 (0.482–0.995)	−0.491	0.210	0.824 (0.525–1.292)	0.500 (0.268–0.934)
Non-TB-related death	0.849 (0.709–1.018)	0.793 (0.659–0.955)	0.209	0.290	0.739 (0.587–0.930)	0.919 (0.672–1.257)

(A) Underweight group id compared to the normal weight group. (B) Overweight group is compared to the normal weight group. BMI, body mass index; cOR, crude odds ratio; CI, confidence interval; aOR, adjusted odds ratio; Coef, coefficient; AFB, acid-fast bacilli; TB, tuberculosis. The odds ratios for each outcome were calculated using logistic regression. Multivariate analysis is adjusted for age, sex, current smoking status, heavy alcohol intake, and the presence of underlying comorbidities. Reference to normal weight (18.5 kg/m^2^ ≤ BMI < 23 kg/m^2^).

* Interaction analysis between BMI and sex.

## Discussion

To our knowledge, this is the largest observational study using a prospectively collected nationwide database to evaluate the impact of underweight and overweight status on the initial disease severity and mortality of pulmonary TB. Using nationwide surveillance data from Korea, we identified the impact of baseline nutrition on the clinical presentation and prognosis in patients with pulmonary TB. A higher BMI was associated with a severe initial clinical presentation and worse prognosis. Compared to patients with a normal weight, those who were underweight had severe baseline disease and higher mortality, and those who were overweight had mild baseline disease and lower mortality. The protective effects of being overweight on TB-related and non-TB-related deaths differed between male and female patients.

We found that not only in low- and middle-income countries but also in high-income countries, such as Korea, undernutrition still existed in the twenty first century, and this deleteriously affected the outcomes of anti-TB treatment. Based on our findings, clinicians should actively assess the nutritional status of patients with pulmonary TB at treatment initiation to determine the risk of unfavorable outcomes, recognize the possible causes of undernutrition, and assist in restoring their underlying nutrition. According to a retrospective observational study in Korea (20), which investigated longitudinal changes in BMI from diagnosis to a 2-year follow-up, the majority of patients were able to recover their body weight during the early phase of anti-TB treatment; however, their BMI at the end of the 2-year follow-up period was still lower than that of the general population. A recent large prospective cohort study in India (13) found that severe undernutrition at treatment initiation and lack of BMI increase during treatment were associated with 4- and 5-fold higher mortality rates, respectively. It is necessary to systematically screen nutritional status at treatment initiation, identify and monitor those with undernutrition, and provide nutritional support to ensure that patients can gain weight.

The detrimental impact of undernutrition on mortality rate was higher in male patients than in female patients in our study. Men are more susceptible to contracting TB and have a more severe clinical presentation and poorer outcomes than women. This sex disparity may be due to a combination of biological, epidemiological, and sociocultural factors. For example, men are more likely to be addicted to tobacco and alcohol, both of which are risk factors for TB development. Because men and women with the same BMI status have different body compositions, men with lower fat mass and less energy reserves at the time of TB diagnosis might be at a greater risk of death than women (21). However, few research studies evaluated sex differences in the context of association between BMI and mortality during TB treatment, which requires further attention.

Obesity, which is frequently accompanied by metabolic syndrome, is typically associated with a higher prevalence of cardiovascular disorders, cancer, and diabetes as well as a shortened life expectancy. In contrast, recent studies (22–24) showed a clear inverse relationship between BMI and TB incidence, which suggests an obesity paradox. According to the recent population-based longitudinal study, higher BMI also reduced long-term mortality among TB survivors (25). However, effect of being overweight on mortality during anti-TB treatment was controversial (7, 15). One of our key findings was the protective impact of being overweight on the clinical presentation and mortality in patients with pulmonary TB, based on the results of our multivariable logistic regression analysis. This phenomenon of better clinical outcomes among obese patients has been frequently observed in many diseases, such as cardiovascular disease (26). Higher daily protein and energy intakes among obese patients, which could improve immune function, could benefit TB outcomes.

It is noteworthy that the protective effects of being overweight on TB-related- and non-TB-related deaths differed between female and male patients. The protective effect of being overweight on TB-related death, which is frequently observed during the early phase of anti-TB treatment, was apparent in female patients. This aligns well with the finding of milder forms of initial clinical presentation in female patients, which could be associated with a lower likelihood of early TB-related death. In male patients, being overweight was associated with a lower rate of non-TB-related death, which occurred during the late phase of anti-TB treatment. There are several common modes of death among patients with non-TB-related conditions, such as acute respiratory failure, cancer, septic shock, and cardiovascular disease, including sudden cardiac death (27). Male patients may benefit more from being overweight in avoiding these complications, which could be ascribed to the obesity paradox (28). Because we used BMI to define overweight status and did not collect other data, we could not identify the mechanisms that could explain our results. Knowledge of other aspects of being overweight and obesity, such as body composition, visceral adiposity, sarcopenic obesity, and cardiac fitness, will help to better explain the relationship between being overweight and TB outcomes.

The key strength of our study is the analysis of a large number of notified TB cases collected systematically across the country, representing the actual TB burden in Korea. Thus, policymakers can use it to prioritize curative and preventive action plans. However, this study has several limitations that should be acknowledged. We could not collect additional laboratory findings, such as blood cell counts and C-reactive protein, albumin, and interferon-gamma levels, owing to the lack of relevant inflammatory biomarkers in the prospective design of a nationwide cohort database. This study was performed in a high-income country with an intermediate TB burden and a low prevalence of human immunodeficiency virus infection, which may limit the generalizability of our findings.

In conclusion, being underweight at treatment initiation was associated with severe clinical presentation and a higher mortality rate among patients with pulmonary TB. In contrast, being overweight was associated with better outcomes, depending on patient sex. Although a large amount of literature supports the importance of controlling undernutrition among patients with TB, it has not been emphasized in the TB world in terms of both research and clinical aspects. Nutritional assessment at the time of TB diagnosis should be incorporated into standard TB care. Furthermore, it is necessary to design and implement appropriate nutritional support programs to improve the treatment outcomes in malnourished patients with TB.

## Data availability statement

The data analyzed in this study is subject to the following licenses/restrictions: The Korea Disease Control and Prevention Agency (KDCA) has the authority to hold and analyze surveillance data for public health and research purposes. The KDCA approved the data use and provided the data without personal identification information. Requests to access these datasets should be directed to H-KK, gusrud9@yahoo.co.kr.

## Ethics statement

The studies involving humans were approved by Institutional Review Board of Ilsan Paik Hospital, Inje University. The studies were conducted in accordance with the local legislation and institutional requirements. The Ethics Committee/institutional review board waived the requirement of written informed consent for participation from the participants or the participants' legal guardians/next of kin because the study analyzed surveillance data for public health and research purposes.

## Author contributions

JM and H-KK conceived the study, performed all analyses, and drafted the manuscript. JSK, SL, and JP supervised the research project. JM, JSK, HK, YK, JO, Y-JJ, EL, BY, KL, JA, JWK, YH, SL, JP, and H-KK collected data. All authors have read and approved the final manuscript.
