# Supplementary material for: Effects of underweight and overweight on mortality in patients with pulmonary tuberculosis
Source: Front Public Health. 2023 Sep 19;11:1236099. doi: 10.3389/fpubh.2023.1236099 (PMC10546415; doi:10.3389/fpubh.2023.1236099)
Supplement: Supplementary file 1 [file Data_Sheet_1.docx]

**Supplemental Table S1.** Frequencies of each initial presenting symptoms in pulmonary tuberculosis patients

| Variables | | Total |
| --- | --- | --- |
|  |  | (n=9,721) |
| Initial presenting symptoms | |  |
|  | Presence of any symptoms | 6,284 (64.6%) |
|  | Cough/phlegm | 4,016 (41.3%) |
|  | Dyspnea | 1,551 (16.0%) |
|  | Chest pain | 579 (6.0%) |
|  | Hemoptysis | 476 (4.9%) |
|  | Fever | 1,225 (12.6%) |
|  | General weakness | 565 (5.8%) |
|  | Weight loss | 805 (8.3%) |

**Supplemental Figure S1**. Proportions of presences of each initial presenting symptoms according to body mass index in enrolled pulmonary tuberculosis patients

**Supplemental Figure S**2. Proportions of positive results of microbiological tests and presences of cavity and bilateral infiltration on chest radiography according to body mass index in enrolled pulmonary tuberculosis patients
